# Collective behaviour in vertebrates: a sensory perspective

**DOI:** 10.1098/rsos.160377

**Published:** 2016-11-16

**Authors:** Diana Pita, Bertrand Collignon, José Halloy, Esteban Fernández-Juricic

**Affiliations:** 1 Department of Biological Sciences, Purdue University, West Lafayette, IN, USA; 2 Université Paris Diderot, Sorbonne Paris Cité, LIED, UMR 8236, 75013 Paris, France

**Keywords:** collective animal behaviour, sensory system, visual field, visual acuity

## Abstract

Collective behaviour models can predict behaviours of schools, flocks, and herds. However, in many cases, these models make biologically unrealistic assumptions in terms of the sensory capabilities of the organism, which are applied across different species. We explored how sensitive collective behaviour models are to these sensory assumptions. Specifically, we used parameters reflecting the visual coverage and visual acuity that determine the spatial range over which an individual can detect and interact with conspecifics. Using metric and topological collective behaviour models, we compared the classic sensory parameters, typically used to model birds and fish, with a set of realistic sensory parameters obtained through physiological measurements. Compared with the classic sensory assumptions, the realistic assumptions increased perceptual ranges, which led to fewer groups and larger group sizes in all species, and higher polarity values and slightly shorter neighbour distances in the fish species. Overall, classic visual sensory assumptions are not representative of many species showing collective behaviour and constrain unrealistically their perceptual ranges. More importantly, caution must be exercised when empirically testing the predictions of these models in terms of choosing the model species, making realistic predictions, and interpreting the results.

## 1. Introduction

For many species, social interactions play an important role in life history, often leading to the formation of social groups [1]. From an individual's perspective, joining groups can be beneficial (i.e. enhancing mating opportunities, food localization, and predator detection [2]). Additionally, many of these benefits depend on the group structure as groups can vary in size (i.e. number of individuals), density (i.e. number of individuals per unit area/volume), spacing (i.e. distance between group mates), shape (i.e. length and width of the group) and polarity (i.e. individuals face similar or different directions, leading to high or low alignment, respectively) [3,4].

One fundamental aspect of group structure is understanding how individuals interact within the group [1], as these interactions can lead to large-scale patterns of coordinated movement, known as collective behaviour [1,5–8]. For example, when group mates vary their distance, position or speed relative to their nearest neighbours, the density and shape of the group can change [1,9,10]. Many fish species are known to organize themselves into two main types of collective states, shoals and schools [11]. Shoals are loose aggregations (i.e. random orientation, alignment and spacing of individuals) often formed during low stress contexts (i.e. foraging). Schools often form when a predator is detected, resulting in individuals maintaining high alignment and close spacing, increasing cohesiveness and consequently, reducing the chances of mortality [11]. Other forms of collective behaviour, particularly in fish, include swarms and toruses [7].

The persistence of collective behaviour relies on individuals being able to acquire, interpret and make use of relevant social information [10,12]. Social information conveys aspects about the sender's current state, encoded in the form of inadvertent cues (e.g. shift in body position of a neighbour) or intended signals (e.g. alarm call) [13]. Considering that information is costly to obtain, species are thought to have evolved sensory systems that may be efficient in acquiring information under particular ecological conditions [5,14]. Therefore, in some species, individuals may rely heavily on certain sensory modalities (e.g. visual system) or certain dimensions within a sensory modality (e.g. visual resolution) to acquire specific types of social information [15–17]. Variations in sensory input can influence the interactions between group members. For instance, studies conducted with saithe (*Pollachius virens*) found that eliminating mechanosensory information decreased neighbour distances and increased the number of collisions between group members, while eliminating visual information led to an increase in neighbour distance [18].

Despite the role that the sensory systems may play in the acquisition of social information, our current understanding of collective behaviour does not generally consider the sensory constraints of different species [19]. In the past, many models used to study collective behaviour followed simplistic assumptions and interaction rules largely based off inanimate particles [20]. Some recent models have considered aspects of sensory systems, such as chemotaxis [21], and vision [22,23]. Despite these attempts, many theoretical models still (i) limit an entire sensory modality to one dimension and do not include key aspects of sensory filtering relative to collective behaviour, (ii) generalize a single sensory configuration to multiple species or (iii) inaccurately represent the sensory system of the study species [23–25]. Establishing the role of different sensory system configurations in collective behaviour from a theoretical perspective can provide novel insights into how sensory filtering may influence some of the mechanisms underlying collective behaviour in different taxa.

In this study, we investigated how sensitive two types of collective behaviour models (metric model (MM) and topological model (TM)) are to the constraints of the sensory system in different species by focusing on one sensory modality (i.e. vision) and assessing the role of two sensory components (i.e. visual coverage and visual acuity). We used models with metric-interaction rules and topological-based rules because they have been used frequently and continue to serve as a foundation for developing new models of collective behaviour [8,26,27]. We established the influence of visual assumptions on the predictions of these models relative to the number of groups in the population, group size (i.e. total number of interacting individuals), polarity (i.e. degree of individual alignment), nearest neighbour distance (i.e. spacing between individuals), speed at which group properties stabilized (i.e. speed of social information transfer) and the internal structural stability of the group (i.e. degree to which individuals maintain their position relative to neighbours in the group). We compared the model outputs based on: (i) a set of visual assumptions used often throughout the literature and applied across species despite their likely very different sensory configurations (hereafter, classic assumptions), and (ii) a set of assumptions from direct measurements of the visual systems of four vertebrates (two fish and two birds) that engage in collective behaviour and predominantly use vision during social interactions (hereafter, realistic assumptions).

## 2. Visual sensory assumptions in collective behaviour

In visually oriented species, visual coverage and visual acuity are two components of the visual sensory dimension that can mediate social interactions. For example, an individual's capacity to detect the presence of surrounding neighbours is influenced by the degree of visual coverage or visual field (i.e. the inverse of its blind area at the rear of the head [28]), while the ability to resolve subtle differences in neighbour identity and behaviour at a given distance is dependent upon the spatial resolving power of the retina (i.e. visual acuity) [29].

Collective behaviour models tend not to be species-specific when they attempt to incorporate vision-based parameters. For example, many models often use a 60° blind area for modelling fish and a 90° blind area for modelling birds [30,31]. The origin of these values can be traced back to measurements obtained from single species through direct observation of group behaviour or inferred indirectly without taking measurements of the projections of the retinal boundaries in space. In the case of fish, blind area values have been obtained from indirect estimates of gadoid fish [31] while visual acuity values (i.e. 0.5–2 body lengths) have been inferred from nearest neighbour distance ranges observed in minnow (*Phoxinus phoxinus*) groups [32–34]. In the case of birds, blind area and visual acuity values were largely inferred without taking direct measurements from any species (e.g. [30]). Additionally, some studies estimated visual coverage from the projections of the eyes [24,25], but this method does not provide precise assessments of the actual projection of the retinal boundaries in space [28].

One of the consequences of using of these inferred visual parameters in collective behaviour models is that the outputs may be constrained to the same type of sensory filtering. However, there is a large amount of between-species variation in the configuration of the visual system [35–39]. Additionally, it is unclear whether these classic sensory parameters used in models accurately represent all the species within each respective taxonomic group. For example, one avian species often used in collective behaviour research, the European starling (*Sturnus vulgaris*) has a blind area of 64° [40], which is 26° lower than the assumed 90° blind area used to model its flocking behaviour [30,41]. Golden shiners (*Notemigonus crysoleucas*) and zebrafish (*Danio rerio*) have blind areas of only 21° and 25° [42], respectively, which is about half the values commonly used in studies that model fish collective behaviour[33,43–45]. Employing the same set of sensory assumptions across species could potentially impair our ability to (i) make informed predictions about collective behaviour in different taxa, (ii) choose the right species to empirically test those predictions (i.e. species whose sensory configuration matches the model assumptions), (iii) interpret the mismatches between theoretical predictions and empirical data, and ultimately (iv) fail to isolate the mechanisms driving group member interactions.

## 3. Material and methods

The MM and TM we used belong to the family of individual-based models, which provide a way of understanding the mechanisms that lead to different emerging group configurations [1,46]. Additionally, individual-based models allow scientists to compare the output of simulations in which agents that follow different local interaction rules [26,47]. The metric-based interaction rules [33,48] define three metric zones that radiate from the centre of the individual: (i) repulsion, (ii) alignment, and (iii) attraction. Neighbours located within the repulsion zone are avoided. Neighbours located within the alignment and attraction zones are used to determine the individual's successive movement decisions, either by matching its heading to that of its neighbours or moving closer to them [7].

Models that follow topological-based rules were introduced after empirical evidence showed that starlings respond to a set number of neighbours independent of their metric neighbour distance [8]. Topological-based rules allow individuals to weigh information equally from a set number of near or distant neighbours while incorporating similar principles of attraction, repulsion and alignment [27].

We studied the effects of two components of the visual system: visual coverage and visual acuity, and incorporated both as the basis for the realistic assumptions. We operationally defined visual coverage as the area around an individual's head along the horizontal plane of the body from which it can gather visual information [28], and visual acuity as the maximum distance that an individual can spatially resolve social cues, which can be used as a proxy for an individual's social interaction range [29,42]. Our proxy of visual acuity is not the maximum distance an individual can perceive the whole body of a conspecific, but rather the distance it can resolve specific morphological cues (i.e. head height for birds and body height for fish) that would lead to the recognition of a member of the same species. Consequently, our proxy of visual acuity is assumed to more closely resemble the distance that animals react to neighbours. In the electronic supplementary material, we explain how we calculated the interaction range of each species using each species' anatomically derived visual acuity and visual coverage to generate the realistic assumptions.

As study systems, we used two bird species from the Order Passeriformes (Family Sturnidae: European starling *S. vulgaris*; Family Icteridae: red-winged blackbird *Agelaius phoeniceus*) and two fish species from the order Cypriniformes (Family Cyprinidae: golden shiner *N. crysoleucas* and zebrafish *D. rerio*) (table 1). These species engage in social interactions using visual cues [50–53], and have measured descriptions of their visual system configurations [40,42,49] (E Fernández-Juricic 2016, unpublished data). Additionally, some of these species have empirical data available on their grouping behaviour (table 2), which we used to increase the level of species-specificity in the models.

**Table 1.** Comparison of the classic visual assumptions assumed throughout the literature for birds and fish along with the realistic visual assumptions determined physiologically from species representatives (i.e. European starling, red-winged blackbird, golden shiner and zebrafish). Visual coverage corresponds to regions of the visual field configuration, excluding the blind area in the horizontal plane of the head. The visual acuity reflects the minimum visual acuity necessary to resolve social cues from conspecifics (i.e. head for birds and body for fish) of each species represented in units of body length (BL) (see electronic supplementary material). Parameters applied to the interaction rules of both MM and TM.

	classic visual assumptions		realistic visual assumptions	
species	visual coverage	visual acuity	visual coverage	visual acuity
European starling	270° [30]	6 BL [8]	296° [40]	15.5 BL [49]
Red-winged blackbird			314°^a^	19.5 BL^a^
golden shiner	300° [31]	2 BL [32]	335° [32]	16.5 BL [32]
zebrafish			339° [42]	7.7 BL [42]

^a^E Fernández-Juricic 2016, unpublished data.

**Table 2.** Species assumptions that remained constant across both models and sets of visual assumptions (i.e. classic versus realistic). Values obtained from the literature according to: group size (number of individuals in the group), speed (body lengths per second, BL s^−1^), minimum separation distance in terms of body length (BL) and the maximum turning angle per each species measured in degrees (see electronic supplementary material), analysed from video (electronic supplementary material) [49].

species	group size	speed	minimum separation	maximum turning angle
European starling	30 [54]	50 BL s^−1^ [55]	1 BL [8]	131° [49]
red-winged blackbird	100 [56]	40 BL s^−1^ [57]	1 BL [56]	132° [49]
golden shiner	45 [58]	1 BL s^−1^ [59]	1 BL [60]	134° [49]
zebrafish	10 [61]	5 BL s^−1^ [62]	1 BL [63]	87° [49]

We examined the following group parameters: (i) number of groups in the population, (ii) average group size (i.e. the average number of individuals per each group calculated from the total simulated population), (iii) the polarity (i.e. degree to which all individuals in the population are facing in the same direction), (iv) the average nearest neighbour distance between all individuals in the population, (v) speed at which the group stabilized and (vi) the internal structural stability of the group. A group was defined as a subset of individuals that interact with at least one other member within this subset. Polarity was computed as

$$P(t)=\frac{1}{N}\left|\overset{N}{\underset{i=1}{\sum }}v_{i}(t)\right|,$$

with *P*(*t*) the polarity of the group at time step *t*, *N* the total number of agents and *v*_i_(*t*) the unit direction vector of the agent *i* at time step *t*.

We assessed the time it took for the group to stabilize into its final configuration for certain group properties (i.e. number of groups, group size, polarity and nearest neighbour distance), which we associated to the speed of information transfer within the group. The lapse of time to stabilization can be regarded as a proxy of the speed of social information transfer in the group. For example, it has been shown that species with greater perceptual ranges (i.e. higher visual acuity) are capable of detecting social cues from farther away, which can potentially enhance the speed at which information travels through the group [29]. The speed of information transfer is a relevant component to collective interactions because it indicates how efficiently individuals uptake social information and modify their behaviour [14], ultimately leading to the final group configurations.

Finally, we examined the spatial distribution of neighbours surrounding an individual in order to determine the probability of neighbour presence. This parameter provides an indication of the internal structural stability of a group (i.e. higher peak neighbour probability values are associated with more stable groups). To calculate the peak neighbour probability, we computed a normalized two-dimensional histogram of the positions of the neighbours surrounding each individual at each time step of the simulation. We translated and rotated the positions of all of the individuals to bring the focal agent towards the origin of the two axes, oriented towards the zenith (i.e. 90° uppermost region of the virtual environment). We measured the positions of all neighbours and sorted them in a two-dimensional normalized histogram that provided the probability to find a neighbour in a specific position of the space surrounding the focal individual.

We coded a self-propelled agent model to simulate groups of virtual agents that moved on a two-dimensional torus surface of size *L*^2^, with *L* = 43 [20]. The agents interacted with each other during collective motion. At each time step, the agents perceived other individuals in their perceptual range and updated their position according to the neighbours' position and orientation. The perceptual range of an agent was characterized by an interaction zone delimited by (i) a ‘vision-cone’ parameter and (ii) a ‘vision-distance’ parameter. The ‘vision-cone’ can be thought of as the individual's visual field (excluding the blind area), and the ‘vision-distance’ was represented as the maximum metric distance that an individual can interact with its neighbours (i.e. a proxy of visual acuity). For example, if a group member was beyond the ‘vision-distance’ threshold of the focal individual, it was not considered in its future movement decisions. In addition to their perceptual range, the agents were also surrounded by a short-range repulsion zone (i.e. 1 body length) and tended to avoid the conspecifics situated in this zone.

The agents updated their positions *X_i_* with a velocity vector *V_i_* though a discrete time process:

$$X_{i}(t+\delta t)=X_{i}(t)+V_{i}(t)\delta t$$

and

$$V_{i}(t+\delta t)=v_{i}(t+\delta t)\Theta_{i}(t+\delta t),$$

with *v_i_* representing the linear speed of the agent *i* and *Θ_i_*, its orientation. In our simulations, the linear speed of the agent was constant and species-specific (table 2). The orientation was computed according to the position and orientation of the neighbours. To determine the subset of neighbours that influenced the next orientation of the focal agent, we implemented two different methods: the metric perception approach and the topological perception approach. In the metric perception approach, all individuals situated in the perception zone of the focal agent influenced its future orientation. In the topological perception approach, only the *n* proximate agents in the perception zone influenced the focal agent's future orientation, with *n* being species-specific. Once this subset of neighbours was identified, the model evaluated the presence of neighbours in the repulsion zone of the focal agent. If a neighbour was detected in this zone, the focal agent updated its positions by an interaction vector pointing in the opposite direction of the detected individual. However, if no neighbours were detected in the repulsion zone, then the focal agent would only interact (i.e. move towards and align) with neighbours located in other regions of its perception zone [7].

For each species, we ran 50 simulations of 3600 time steps with each perception approach (metric or topological) and each set of parameter values (classic or realistic) for each of the four studied species.

## 4. Results

**Figure 1.**
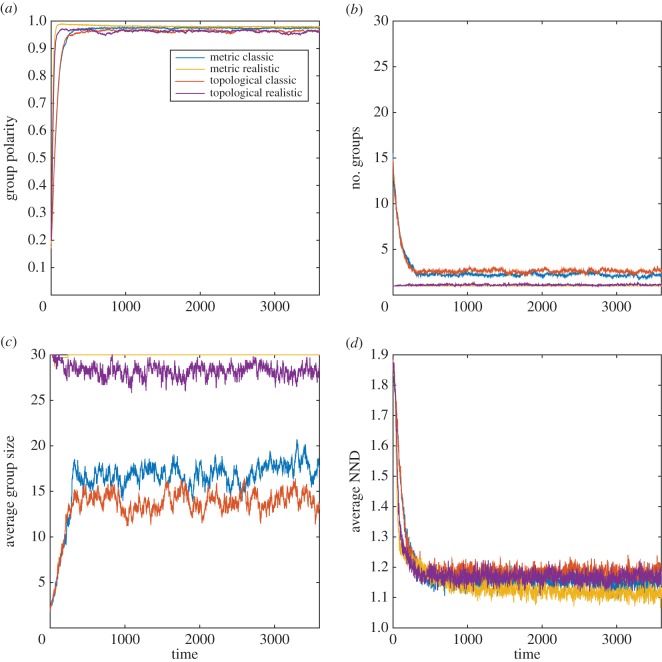
MM and TM outputs comparing the group properties: (*a*) group polarity, (*b*) number of groups, (*c*) average group size and (*d*) average nearest neighbour distance (NND) for the classic and realistic visual assumptions in the European starling.

**Figure 2.**
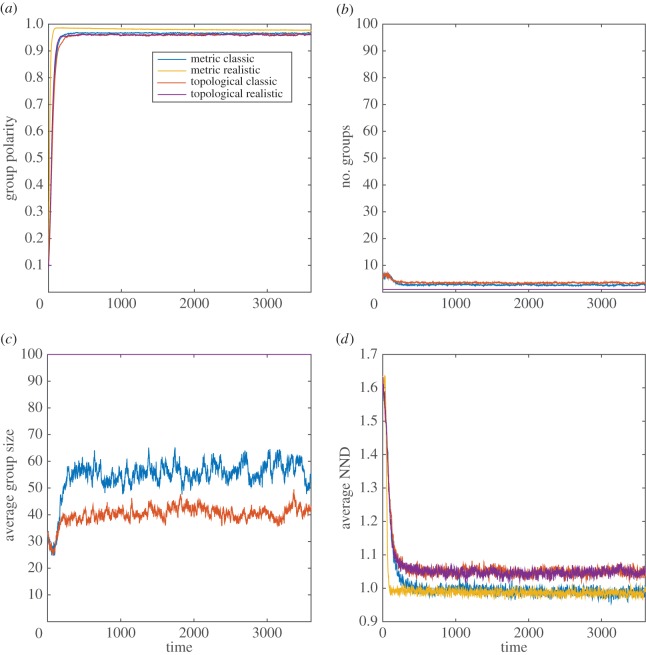
MM and TM outputs comparing the group properties: (*a*) group polarity, (*b*) number of groups, (*c*) average group size and (*d*) average nearest neighbour distance (NND) for the classic and realistic visual assumptions in the red-winged blackbird.

**Figure 3.**
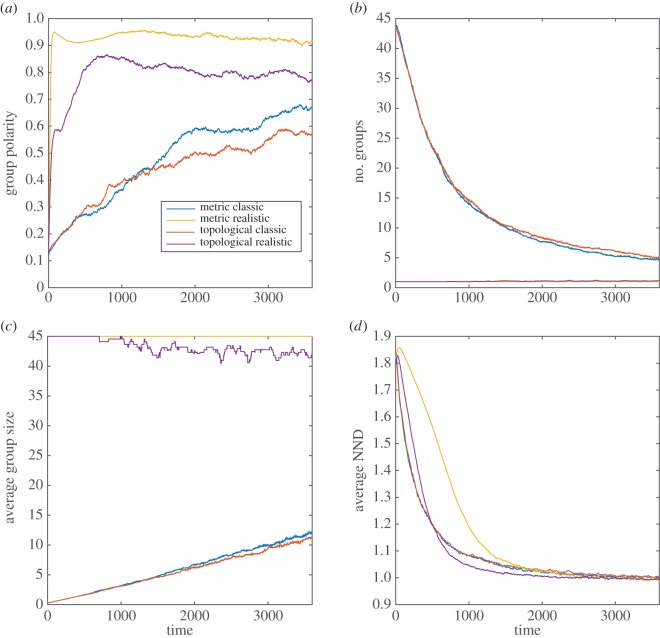
MM and TM outputs comparing the group properties: (*a*) group polarity, (*b*) number of groups, (*c*) average group size and (*d*) average nearest neighbour distance (NND) for the classic and realistic visual assumptions in the golden shiner.

**Figure 4.**
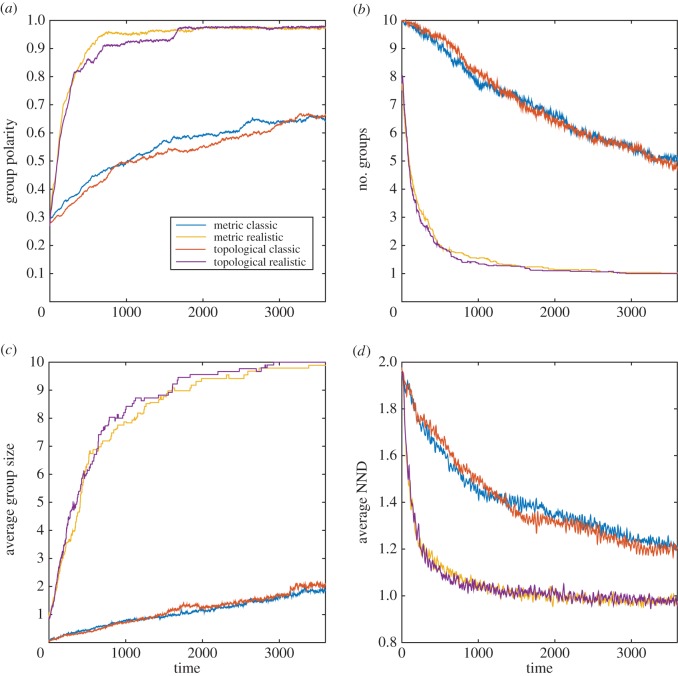
MM and TM outputs comparing the group properties: (*a*) group polarity, (*b*) number of groups, (*c*) average group size and (*d*) average nearest neighbour distance (NND) for the classic and realistic visual assumptions in the zebrafish.

Overall, the results of the modelling simulations indicate that in many cases, both the MM and TM are sensitive to modifications of the sensory assumptions (figures 1–4; electronic supplementary material, table S1).

In the European starling and the red-winged blackbird, both models yielded several small groups with the classic sensory assumptions compared with the realistic sensory assumptions, which yielded one large group composed of all members of the simulated population (figures 1 and 2; electronic supplementary material, table S1*a,b*). Both models yielded similar polarity and nearest neighbour distance values upon stabilization irrespective of the type of sensory assumption (figures 1 and 2; electronic supplementary material, table S1*a*,*b*). However, for both bird species, it took longer to achieve a stable polarity and nearest neighbour distance in both models with the classic than the realistic sensory assumptions (figures 1 and 2).

In the golden shiner and zebrafish, both models yielded several smaller groups with the classic compared with the realistic sensory assumptions (figures 3 and 4; electronic supplementary material, table S1*c*,*d*). Additionally, both species groups achieved lower polarity and took longer to reach the stable polarity values with the classic than with the realistic sensory assumptions (figures 3 and 4; electronic supplementary material, table S1*c*, *d*). Furthermore, in the golden shiner, both models produced similar results in terms of neighbour distance considering both sensory assumptions (figure 3; electronic supplementary material, table S1*c*). However, in the zebrafish, both models yielded slightly longer neighbour distances with the classic than with the realistic sensory assumptions (figure 4; electronic supplementary material, table 1*d*). Lastly, for both fish species, models with the classic assumptions took a longer period of time to reach the stable neighbour distance values than with the realistic sensory assumptions (figures 3 and 4).

**Figure 5.**
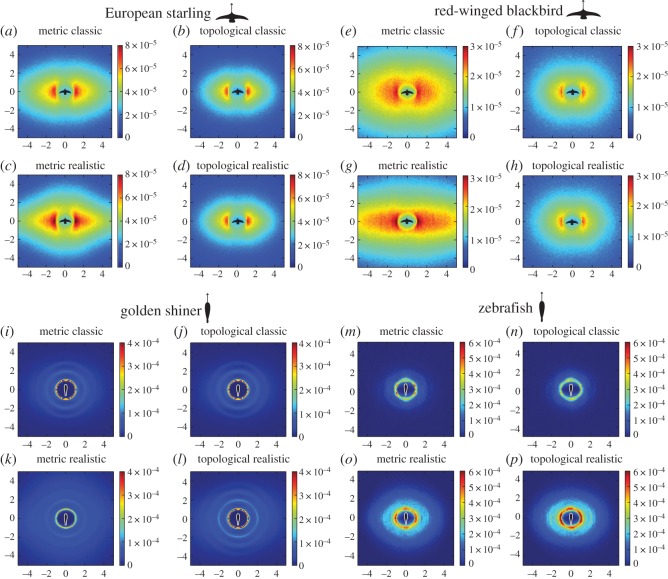
Spatial map illustrating the probability of neighbour presence surrounding an individual (i.e. warmer colours indicate high probability, cooler colours indicate low probability). Values detail the averages per each species represented throughout the entire stimulus duration (3600 time steps).

In terms of the probability of neighbour presence surrounding an individual, we found that in all of the species, the realistic sensory assumptions resulted in groups with higher structural stability. For both fish species, the peak probability of neighbour presence was higher in the TM using realistic sensory assumptions. However, for both bird species, the probability of neighbour presence reached a higher peak probability with the MM using realistic sensory assumptions (electronic supplementary material, figure S1). Additionally, the area encompassing high probability of neighbour presence (red colour in figure 5) tended to be higher in the models with realistic sensory assumptions, particularly for birds.

## 5. Discussion

We found that the classic sensory assumptions often used in collective behaviour models yielded different outcomes in terms of key parameters describing group structure (e.g. mostly group size and total number of groups, and in some cases polarity, neighbour distance, group stability, along with the speed to reach stable values). Additionally, we generated new sets of predictions about collective behaviour based on visual sensory constraints, which can be tested empirically in the future using different methodological approaches.

**Figure 6.**
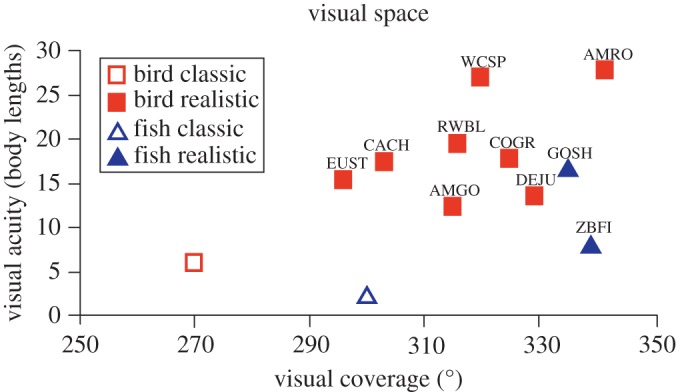
Overlap of the visual space of the visual coverage (measured ophthalmoscopically) and interaction distance (derived from the minimum visual acuity and converted into units of body length) comparing the classic parameters used for birds and fish and the realistic visual parameters of the animals used in this study (European starling (EUST), red-winged blackbird (RWBL), golden shiner (GOSH) and zebrafish (ZBFI)). Additionally, we have included other realistic estimates of bird species for which both the visual field and visual acuity information has been measured (American goldfinch (AMGO), American robin (AMRO), Carolina chickadee (CACH), common grackle (COGR), dark-eyed junco (DEJU), and white-crowned sparrow (WCSP)).

The main reason for the discrepancy between the modelling outcomes is that the classic sensory assumptions appear to substantially underestimate values of visual acuity and visual coverage for both species of birds and fish. Figure 6 shows the different positions of the classic versus the realistic assumptions in a visual space bounded by visual acuity and visual coverage. This figure not only shows the four species used in this study, but also other species that have had both visual parameters measured. Realistic sensory assumptions result in a greater area available for social interactions because species appear to have higher visual coverage and visual acuity than often assumed by collective behaviour models [30,33,34,45].

Our collective behaviour models with realistic assumptions led in general to fewer groups and larger group sizes for both bird and fish species than the models with the classic sensory assumptions. Greater perceptual area ranges can increase the volume of space individuals have to interact with others because of the higher chances of detecting them and resolving their subtle behaviour, thereby increasing the number of neighbours that are taken into consideration in movement decisions. This finding appears to support some empirical evidence showing that groups of golden shiners tend to organize into a single large group, as opposed to assembling into several smaller groups [59]. Furthermore, our results are in agreement with previous modelling studies that explored variations in an individual's perceptual range by altering a single visual dimension, such as visual coverage (i.e. through variations in the width of the blind area). For example, Romey & Vidal [64] found that larger blind areas caused large groups to break up into smaller, more numerous groups with lower density. Similarly, Newman & Sayama [65] found that increasing the size of an individual blind area resulted in the abolishment of certain types of collective behaviour (i.e. milling patterns), and Couzin *et al.* [7] demonstrated that a variety of different types of collective behaviours could be produced by altering the radius of the individual's interaction range (i.e. orientation and attraction).

In both fish species, we also found differences in polarity between models with different sensory assumptions. For example, in both fish species, the model with the realistic sensory assumptions yielded higher polarity values (i.e. group tended to move in the same direction) than the one with the classic sensory assumptions. In both models, a group was defined when two or more individuals interacted with each other. As group members continue to factor in the behaviour of an individual during the running time of the model, all members of the group tended to coalesce into similar directional headings, resulting in an increase in polarity. In the case of the realistic sensory assumptions, where the entire population formed one large group, polarity was very high (i.e. close to 1). However, for the classic sensory assumptions, polarity was comparatively lower due to the multiple, independent groups formed in the population with different directional headings among them. This finding is somewhat supported by empirical evidence. For instance, European starlings have been shown to maintain large flock sizes with high polarity (i.e. greater than 0.9) [66]. Similarly, golden shiner groups also tend to associate in highly polar configurations [59].

The realistic sensory assumptions also predicted fish species to produce groups with slightly shorter neighbour distances compared with the classic sensory assumptions. According to the interaction rules that define the minimum neighbour separation (i.e. repulsion zone), individuals are limited to having separation distance of approximately 1 body length in size [7]. Therefore, as the simulation proceeds, and the group increases in size with the realistic sensory assumptions, individuals would tend to reduce the neighbour distances closer to the 1 body length threshold. On the other hand, for small, sparse groups, like the situation observed with the classic sensory assumptions, the nearest neighbour distance would be larger than 1 body length as individuals are separated farther in space.

We also found that in all of the species considered, models with the realistic assumptions yielded groups with higher internal group stability (i.e. higher peak surrounding neighbour probability), which is probably also a function of the higher perceptual range of the realistic assumptions. Interestingly, groups that achieved the highest stability were obtained with the MM for the bird species and the TM for the fish species. This difference in stability predictions between birds and fish is probably explained by birds having higher average speeds than fish in our simulations (table 2). For instance, by moving at higher speeds, birds can take into account perceptually, and interact with, a greater number of neighbours within the confines of their metric-interaction range. Since the TM limits the total number of neighbours that an individual can interact with, it ends up producing lower peak neighbour probability values than the MM. However, for the fish species, it takes longer for individuals to detect neighbours metrically, probably due to their slower speeds. On the other hand, with the TM, individuals are capable of immediately interacting with neighbours in the population, moving closer to them, even if they are separated farther than the metric distance in space. Therefore, for both fish species, the TM ends up producing more stable groups (i.e. higher peak surrounding neighbour probability values) compared with the MM.

Our models provided a series of very specific predictions about collective behaviour: higher visual acuity and wider visual coverage is expected to lead to lower numbers of groups in the population, each with larger group size, higher polarity, shorter neighbour distance, higher structural stability and a faster spread of social information. These predictions can be tested at the comparative level across species (e.g. birds, fish) with different visual field configuration and/or visual acuity. However, the results of comparative tests may be confounded by other differences between species in ecology, physiology, and degree of shared ancestry. Our predictions could also be tested experimentally within species (i.e. different age classes in some fish, amphibians and reptiles or different mutants in some model organisms like zebrafish) by manipulating visual coverage and visual acuity. Studies on birds have used eye caps to fully or partially occlude vision [67–70]. Therefore, visual coverage could be manipulated by applying eye caps to increase the size of the blind area, and visual acuity could be manipulated by using transparent eye caps with different refractive indices. In fish, a combination of eye caps and artificial lens designs have been used to manipulate various visual dimensions [71], and consequently, these tools can also be used to test our predictions. In model species like zebrafish, genetic mutants may also be used to determine the influence of specific retinal cell types implicated in visual acuity on behaviour. For example, one study examined the foraging success of zebrafish mutants with a reduced population of ultraviolet sensitive cones to assess their ability to visually detect and capture prey when their perception of ultraviolet cues were reduced [72]. A similar approach using zebrafish mutants with decreased retinal ganglion cell populations [73–75] could be used to assess if variations in visual acuity between mutants and wild-type changes the range necessary to perceive conspecific cues.

Current technology is also likely to facilitate the assessment of some of the grouping parameters measured in the model. For example, group size, polarity and neighbour distance can be measured in laboratory conditions with specialized tracking software that can isolate and identify the movements of group members in three dimensions [76,77]. Similarly, sonar technology can be used to measure comparable parameters of large fish schools in the wild [78]. For birds, tracking individual trajectories among large groups in the field is done with small tracking devices, allowing one to estimate group size and neighbour distance [79]. Additionally, stereo photography can also be used to reconstruct the spatial configuration of the flock through time [8], which can estimate group size, neighbour distance, polarity, structural stability and speed of information transfer [8,80].

Our results also have implications for interpreting the results of previous empirical studies by establishing the matching or mismatching of the theoretical predictions versus empirical findings in the light of the sensory assumptions. For example, in zebrafish, it has been found that group sizes in the wild are much larger (i.e. up to 300 individuals, [81]) than group sizes commonly used in laboratory experiments. Our model with the realistic sensory assumptions can be run with realistic group sizes to generate predictions that can then be compared with empirical data. Ultimately, bringing the sensory approach to collective behaviour will allow us to generate species-specific (or genus- or Family- or Order-specific) predictions with more realistic assumptions.

We examined only two properties of the visual system (i.e. visual acuity and visual coverage), but in reality social interactions can be influenced by many other factors (e.g. chromatic contrast, achromatic contrast, position, type and number of centres of acute vision). Some of these factors may actually be quite relevant in collective behaviour. For instance, both MM and TM assume that the quality of visual perception is the same across all regions of the visual field, and consequently, irrespective of the position of the neighbour, the information will have the same weight in influencing the successive movement decisions of the focal individual. In reality, some parts of the visual field have higher acuity than others, because they are subtended in the retina by the centres of acute vision (i.e. areas with high density of photoreceptors, such as the fovea, area [38,82]).

The implication is that neighbours that are visually tracked with the centre of acute vision may have a higher weight in the movement decisions of focal individuals than neighbours tracked with the peripheral area of the retina (i.e. lower visual acuity). For instance, golden shiners typically maintain close distances with neighbours located directly ahead of them (i.e. 60° bearing angle) [77] (see electronic supplementary material, figure S2), and they seem to use their centre of acute vision (an area that projects in approximately the same direction) to track neighbours visually [42]. On the other hand, in European starlings and homing pigeons, nearest neighbours are often located directly adjacent to the forward direction of motion of the focal individual (i.e. 90° bearing angle) [8,83]. However, the projection of the centres of acute vision along the horizontal plane occurs more forward (i.e. 60° for starlings, 66° for pigeons) [53,84], suggesting that these birds are not tracking neighbours with their centres of acute vision (see electronic supplementary material, figure S2). Although some models have aimed to incorporate the idea of neighbours having different weight in the decision-making of the focal individual [19,85,86], none have done so in the context of the centres of acute vision and their position in visual space.

By challenging previous sensory assumptions, our results suggest that the sensory system is a relevant component of collective interactions, at least from a theoretical perspective. Our findings have important implications for future theoretical and empirical collective behaviour research. First, classic visual assumptions are not necessarily representative of the visual system of the species that show some form of social behaviour. Second, classic visual assumptions unrealistically constrain the social visual space (i.e. the area around an individual from which it can get information about group mates) that animals have to coordinate their movements with group mates. Third, not all sensory configurations may be well represented by the same model. For instance, the metric-based interaction rules, in which an individual responds to all neighbours within a defined concentric range, may accurately reflect the visual limitations of species with narrow visual coverage and reduced acuity. However, a species with high visual coverage and acuity may instead follow topological rules where instead of responding to all neighbours within a particular zone, individuals respond to a set number of neighbours regardless of their separation distance [3].

## Supplementary Material

Estimation of species parameters/Detailed results of modeling exercises/Comparison of visual parameters with empirical findings

## Data accessibility

Simulation data are available in the Dryad Digital Repository: http://dx.doi.org/10.5061/dryad.r1921 [87].
