# Supplementary material for: Collective behaviour in vertebrates: a sensory perspective
Source: R Soc Open Sci. 2016 Nov 16;3(11):160377. doi: 10.1098/rsos.160377 (PMC5180114; doi:10.1098/rsos.160377)
Supplement: Estimation of species parameters/Detailed results of modeling exercises/Comparison of visual parameters with empirical findings [file rsos160377supp1.docx]

**Supplementary Material**

**Section 1: Estimation of species parameters: maximum turning angle, interaction distance and projection of the center of acute vision.**

Estimation of species turning angle

Species turning angles were measured using the video analysis software, Tracker (<http://physlets.org/tracker/>). Using recorded videos of live species groups, we randomly selected a total of six individuals and measured their maximum turning angle per second. The measurements of each of these six individuals were then averaged across the group and were then used to represent each species respectively (Table 1b).

Estimation of species interaction distances

We define each species interaction distance as the maximum resolvable distance that an individual was capable of perceiving visual social cues (i.e., head height for birds and body height for fish). We used these features as, in birds, the head region has been shown to provide reliable cues to assess conspecifics during social interactions [1], while in fish, visibility of the body is also likely to provide important information thought to influence social interactions [2].

First, we used the following equation to determine the retinal magnification factor (RMF) [3], which represents the amount of space that an object takes up on the retina, where PND represents the posterior nodal distance, represented as 0.6 multiplied by the axial length of the eye for birds [4], and 2.55 multiplied by the radius of the lens for fish [5]:

$$RMF=\frac{2\pi x PND}{360}$$

After the RMF was determined, we calculated the spatial resolving power to estimate each species’ ability to resolve conspecific features (i.e., head height for birds, body height for fish), using the minimum retinal ganglion cell density (cells/mm^2^) across the retina (D) [6]. We used the minimum as opposed to the maximum retinal ganglion cell density because this value represented the minimum threshold limit allowing an individual to perceive social cues across all regions of its visual field, and not just localized regions of high visual acuity (i.e., center of acute vision). Although, it should be mentioned that the visual acuity achieved with the center of acute vision is much higher than the estimate used in this modeling exercise.

$$SRP= \frac{\mathrm{RMF}}{2} \sqrt{\frac{2D}{\sqrt{3}}}$$

The maximum resolvable visual distance (d) was then calculated using the equation where r represents the radius of the object (i.e., head height for birds and body height for fish) while $\alpha$ represents the inverse of spatial resolving power (SRP) [7]. This distance estimate assumes maximum visual contrast between the object and the background. The final resolvable distance estimate was then converted into units of body length, specific to each species and applied to both models (Table 1a).

$$d= \frac{r}{\tan\frac{\alpha}{2}}$$

**Section 2: Results of the modeling exercises**

Results for both the metric and topological model outputs comparing the group properties: group polarity (i.e., degree of alignment of all individuals in the population), number of groups, average group size (e.g., average number of individuals per each group) and average nearest neighbor distance (NND) for the classic and realistic sensory assumptions. Table 1 depicts the results upon final group stabilization. Additionally, figure 1 illustrates the peak probability of neighbor presence used to assess group stability.

Across all species, the realistic sensory assumptions result in fewer groups composed of a greater number of individuals compared to the classic sensory assumptions upon stabilization (Table 1). For some species (i.e., golden shiner and zebrafish) the realistic sensory assumptions produce populations with higher polarity and slightly shorter nearest neighbor distances upon stabilization (Table 1 c,d). However, across all species, the realistic sensory assumptions produced a faster onset to group stabilization for all measured parameters .

**Table and Figure Legends**

Table 1. Results comparing the classic and realistic visual assumptions for the a. European starling, b. red-winged blackbird, c. golden shiner and d. zebrafish. Values indicate the grouping parameters (i.e., group polarity across the population, number of groups, average group size and average nearest neighbor distance (NND)) that resulted from each model (i.e., metric (MM) and topological (TM) upon stabilization (± SD).

Figure 1. Graph depicting the peak probability of neighbor presence for all species using both the metric and topological versions of the model.

Table 1

a. European Starling

|  | Classic (MM) | Realistic (MM) | Classic (TM) | Realistic (TM) |
| --- | --- | --- | --- | --- |
| Group Polarity | 0.97 ± 0.01 | 0.97 ± 0.005 | 0.96 ± 0.02 | 0.96 ± 0.03 |
| Number of Groups | 2.16 ± 1.07 | 1 ± 0 | 2.64 ± 1.08 | 1.14 ± 0.35 |
| Average Group Size | 17.41 ± 8.71 | 30 ± 0 | 13.63 ± 5.82 | 28.26 ± 4.62 |
| Average NND | 1.14 ± 0.12 | 1.13 ± 0.11 | 1.18 ± 0.14 | 1.17 ± 0.11 |

b. Red-winged Blackbird

|  | Classic (MM) | Realistic (MM) | Classic (TM) | Realistic (TM) |
| --- | --- | --- | --- | --- |
| Group Polarity | 0.97 ± 0.006 | 0.97 ± 0.005 | 0.96 ± 0.010 | 0.96 ± 0.010 |
| Number of Groups | 2.76 ± 1.15 | 1 ± 0 | 3.50 ± 1.29 | 1 ± 0 |
| Average Group Size | 54.35 ± 23.45 | 100 ± 0 | 42.61 ± 20.21 | 100 ± 0 |
| Average NND | 1.00 ± 0.055 | 0.99 ± 0.046 | 1.05 ± 0.057 | 1.05 ± 0.073 |

c. Golden Shiner

|  | Classic (MM) | Realistic (MM) | Classic (TM) | Realistic (TM) |
| --- | --- | --- | --- | --- |
| Group Polarity | 0.67 ± 0.19 | 0.91 ± 0.056 | 0.57 ± 0.24 | 0.77 ± 0.25 |
| Number of Groups | 4.64 ± 1.35 | 1 ± 0 | 5.02 ± 1.72 | 1.14 ± 0.35 |
| Average Group Size | 12.37 ± 4.58 | 45 ± 0 | 11.22 ± 5.51 | 41.85 ± 7.88 |
| Average NND | 1.00 ± 0.025 | 0.98 ± 0.052 | 1.00 ± 0.024 | 0.99 ± 0.014 |

d. Zebrafish

|  | Classic (MM) | Realistic (MM) | Classic (TM) | Realistic (TM) |
| --- | --- | --- | --- | --- |
| Group Polarity | 0.64 ± 0.66 | 0.97 ± 0.01 | 0.66 ± 0.24 | 0.98 ± 0.01 |
| Number of Groups | 5.1 ± 1.62 | 1.0 ± 0.14 | 4.8 ± 1.85 | 1.0 ± 0 |
| Average Group Size | 1.9 ± 0.97 | 9.9 ± 0.77 | 2.1 ± 1.3 | 10.0 ± 0 |
| Average NND | 1.2 ± 0.17 | 0.97 ± 0.10 | 1.2 ± 1.6 | 0.96 ± 0.06 |

Figure 1.


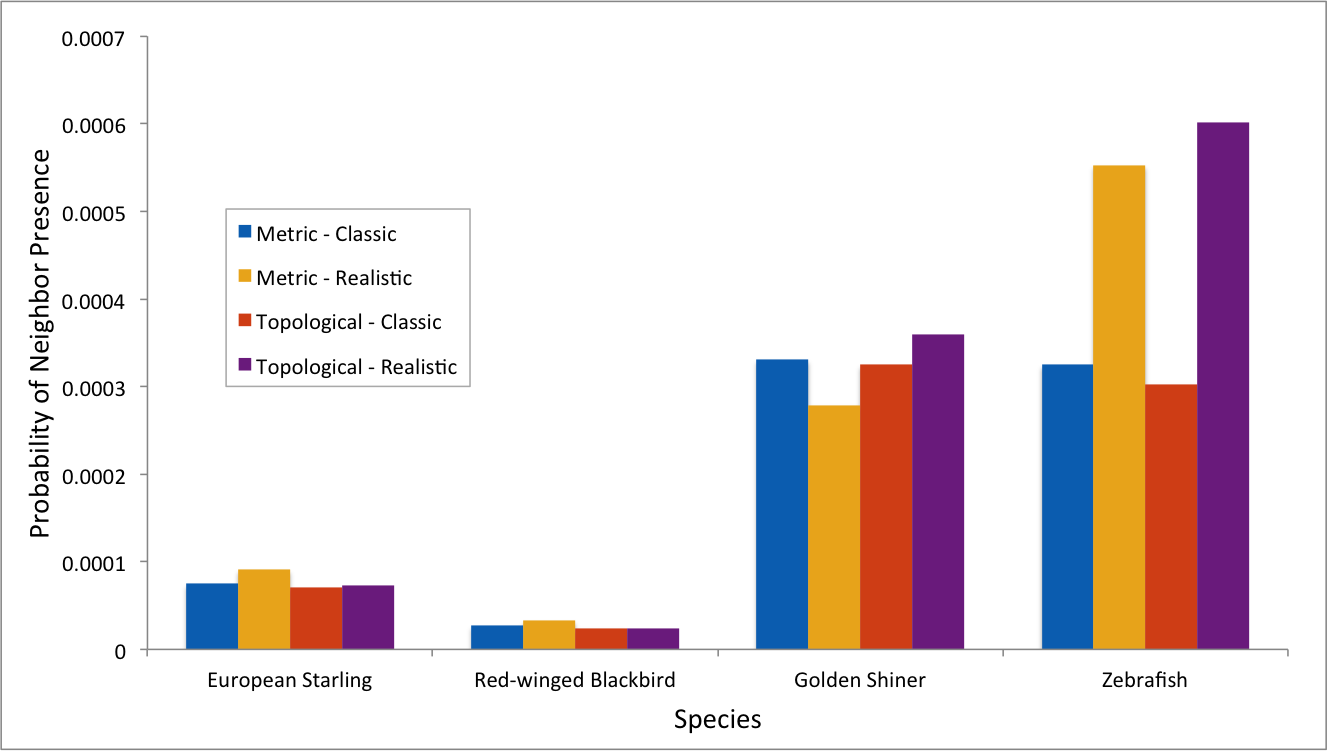


**Section 3: Comparison of visual parameters with empirical findings**

We estimated the projection of the center of acute vision (i.e., visual axis) in the horizontal plane for species in which the angle of the nearest neighbor’s position (i.e., bearing angle) has been measured (Figure 2). European starlings have a 90° bearing angle [8] and their center of acute vision projects 60° from their directional heading [1] (Figure 2a). However, in the golden shiner, the bearing angle and projection of the center of acute vision are 60° [9] and 67° degrees [10] from their directional heading respectively (Figure 2b).

**Figure Legend**

Figure 2. Schematic representation comparing the typical configuration of an individual in a group along the horizontal plane of the body. The angle of the nearest neighbor’s position (i.e., bearing angle) and the of the projection of the center of acute vision (i.e., visual axis) are represented on the focal individual depicted in black, while its nearest neighbor is depicted in grey. a. European starlings have a 90° bearing angle and 60° visual axis from their forward directional heading and b. golden shiners have a 60° bearing angle and 67° visual axis.

Figure 2.

**
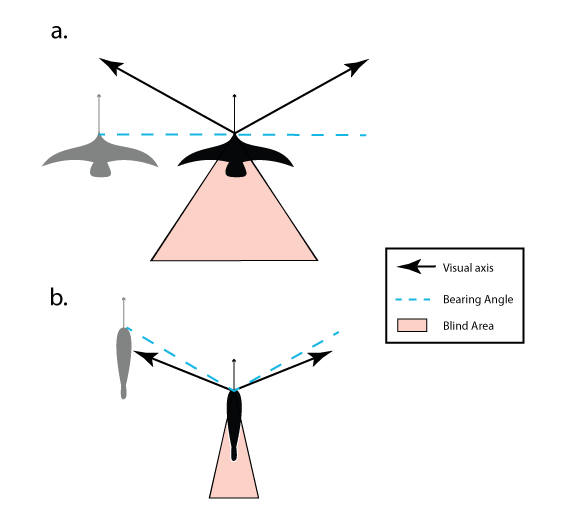
**

References:

1. Butler, S. R. & Fernández-Juricic, E. 2014 European starlings recognize the location of robotic conspecific attention. *Biol. Lett.* **10**, 20140665.

2. Croft, D. P., James, R., Ward, A. J. W., Botham, M. S., Mawdsley, D. & Krause, J. 2005 Assortative interactions and social networks in fish. *Oecologia* **143**, 211–219. (doi:10.1007/s00442-004-1796-8)

3. Pettigrew, J. D., Dreher, B., Hopkins, C. S., McCall, M. J. & Brown, M. 1988 Peak density of ganglion cells in the retinae of michiropteran bats: implications for visual acuity. *Brain. Behav. Evol.* **32**, 39–56.

4. Hughes, A. 1977 The topography of vision in mammals of contrasting life style: comparative optics and retinal organization. In *The visual system in vertebrates*, pp. 613–756. New York: Springer.

5. Collin, S. P. & Pettigrew, J. D. 1988 Retinal ganglion cell topography in teleosts: a comparison between Nissl-stained material and retrograde labelling from the optic nerve. *J. Comp. Neurol.* **276**, 412–422. (doi:10.1002/cne.902760306)

6. Williams, D. R. & Coletta, N. J. 1987 Cone spacing and the visual resolution limit. *J. Opt. Soc. Am. A* **4**, 1514–1523.

7. Tyrrell, L. P., Moore, B. A., Loftis, C. & Fernández-Juricic, E. 2013 Looking above the prairie: localized and upward acute vision in a native grassland bird. *Sci. Rep.* **3**, 1–6. (doi:10.1038/srep03231)

8. Ballerini, M. et al. 2008 Interaction ruling animal collective behavior depends on topological rather than metric distance: evidence from a field study. *Proc. Natl. Acad. Sci. U. S. A.* **105**, 1232–1237. (doi:10.1073/pnas.0711437105)

9. Katz, Y., Tunstrøm, K., Ioannou, C. C., Huepe, C., Couzin, I. D., Tunstrom, K., Ioannou, C. C., Huepe, C. & Couzin, I. D. 2011 Inferring the structure and dynamics of interactions in schooling fish. *Proc. Natl. Acad. Sci.* **108**, 18720–18725. (doi:10.1073/pnas.1107583108)

10. Pita, D., Moore, B. A., Tyrrell, L. P. & Fernández-Juricic, E. 2015 Vision in two cyprinid fish: implications for collective behavior. *PeerJ*
